# Spatial Context of Immune Checkpoints as Predictors of Overall Survival in Patients with Resectable Colorectal Cancer Independent of Standard Tumor–Node–Metastasis Stages

**DOI:** 10.1158/2767-9764.CRC-24-0270

**Published:** 2024-11-26

**Authors:** Hao Kong, Qingxin Yang, Chunwei Wu, Xiangji Wu, Xinrui Yan, Li-Bin Huang, Lu Chen, Zong-Guang Zhou, Ping Wang, Hong Jiang

**Affiliations:** 1 Department of Pancreatic Surgery, State Key Laboratory of Biotherapy, West China Hospital, Sichuan University, Chengdu, China.; 2 Laboratory of Digestive Surgery, State Key Laboratory of Biotherapy, Sichuan University, Chengdu, China.; 3 Key Laboratory of Birth Defects and Related Diseases of Women and Children of MOE, Department of Laboratory Medicine, State Key Laboratory of Biotherapy, West China Second University Hospital, Sichuan University, Chengdu, China.

## Abstract

Although immune checkpoint blockade therapy has shown promising results in a small subset of patients with colorectal cancer with high microsatellite instability, the majority of patients with colorectal cancer do not respond to immune checkpoint blockade therapy. The main obstacle to the success of immunotherapy in cancer treatment is the exhaustion of tumor-infiltrating lymphocytes (TIL). Elucidating the spatial organization of immune checkpoints within the tumor microenvironment (TME) could pave the way for the development of novel prognostic tools and therapeutic strategies to enhance antitumor immune responses. To clarify the spatial and functional diversity of TILs in the colorectal TME, we performed multiplexed IHC to examine the exhaustion of TILs in the TME, the expression of PD-1 and T-cell immunoglobulin and mucin domain–containing protein 3 (TIM-3), which are major biomarkers of T-cell exhaustion, and least absolute shrinkage and selection operator method–Cox analyses of the correlation between colorectal cancer prognosis and TME features. For proof of concept, the antitumor efficacy of TIM-3 and PD-1 dual blockade in colorectal cancer was further evaluated in a CT26 s.c. tumor model of human colorectal cancer. We found that the spatial context of PD-1 and TIM-3 successfully predicted the overall survival of patients with colorectal cancer independent of tumor–node–metastasis stage. Dual targeting of PD-1 and TIM-3 in mouse tumor models inhibited tumor progression and reduced T-cell exhaustion, indicating a potential strategy for improving the clinical treatment of colorectal cancer.

**Significance:**

The identification of specific spatial patterns of immune checkpoint expression that correlate with overall survival in patients with colon cancer suggests a potential prognostic tool for risk stratification and treatment selection. These findings pave the way for the development of novel therapeutic strategies to enhance antitumor immune responses.

## Introduction

Colorectal cancer is the third most common cancer in the world, with increasing morbidity and mortality (1, 2). Recent studies have suggested that the tumor immune microenvironment (TIME) plays a pivotal role in colorectal cancer progression and is considered an important prognostic biomarker (3–6). For example, the immune score, which is based on the CD8/CD3 ratio in the tumor microenvironment (TME), has achieved effective results in predicting the prognosis of patients with colorectal cancer (7). However, the profiles of immune cells in the colorectal cancer microenvironment and their interactions with clinical outcomes have not been clearly established, which warrants further investigation.

Therefore, multiplex IHC (mIHC) has recently been applied to explore the TIME in head and neck tumors, pancreatic cancer, and lung cancer (8–10). By taking advantage of this state-of-the-art technology, we can perform *in situ* analysis of the spatial distribution characteristics of each subgroup of immune cells. The application of mIHC also allows investigation of the interplay of tumor cells and immune cells on a single slide, which provides a new strategy for in-depth study of the TIME. With the emergence and development of mIHC staining techniques, more comprehensive information about immune cell subgroups can be obtained.

In this study, we used mIHC techniques to develop a more refined and personalized new risk prediction system for colorectal cancer. Commonly, the least absolute shrinkage and selection operator method (LASSO) is used to select significant variables from high-dimensional marker data (11, 12). The LASSO‒Cox proportional hazards model was then applied for complex, multifactor survival analyses (12). We constructed a mIHC-based risk score system incorporating 21 spatial pathologic features, covering most of the significant immunologic and pathologic features, to predict survival outcomes. Nomograms were also established to increase the clinical utility of the risk score system for predicting outcomes. As a proof of concept, mouse models of human colorectal cancer demonstrated the antitumor effect of co-blocking TIM-3 and PD-1 by synergistically reversing the exhaustion of T cells.

## Materials and Methods

### Study population and tumor specimen collection

We collected data from patients with histologically confirmed colorectal cancer who underwent curative surgical resection at the Department of Gastrointestinal Surgery, West China Hospital, Sichuan University, China, between March 2009 and December 2016. The project was approved by the West China Hospital Medical Committee (2020-374). All patients provided written informed consent. The included patients had not received any type of neoadjuvant therapy, radiotherapy, targeted therapy, or immunotherapy before surgery. The clinical and pathologic parameters of the included patients were obtained from the patient’s electronic medical records. For this study, all the specimens were regraded according to the eighth edition of the American Joint Committee on Cancer tumor–node–metastasis (TNM) grading system by a pathologist. Clinical follow-up was updated in November 2020. Tissue microarrays (TMA) were constructed for the collected samples. In standard paraffin sections, the tumor regions were histomorphologically analyzed. TMAs were prepared with a 1 mm tissue core and 5 μm thickness via standard procedures. One representative core from primary tissue was used to construct TMAs for each patient.

### IHC staining

All involved tissue samples were processed into paraffin, and paraffin-embedded tissues were cut into 5 μm pieces. For IHC, the slides were immersed in antigen retrieval buffer (GeneTech), and antigen retrieval was then performed in a microwave oven for 20 minutes. Then, 2% BSA (BioFroxx) was used for blocking for 10 minutes, and primary antibodies (details of the antibodies are described in Supplementary Table S1) were diluted in PBS containing 1% BSA and applied overnight. A secondary antibody (Thermo Fisher Scientific) was added after the slides were washed for 30 minutes. After the slides were washed, 3,3'-Diaminobenzidine solution (GeneTech) was added for coloring. Counterstaining was performed via hematoxylin, and the slides were immersed in xylene after dehydration in an ethanol gradient. Finally, the slides were mounted with neutral balsam. The slides were scanned at 20× magnification using a PANNORAMIC 250 scanner (3D HISTECH).

### mIHC

mIHC staining was performed using an Opal 7-color kit (Akoya Biosciences). Briefly, the sections were dewaxed with xylene for 20 minutes. Then, ethanol was used for rehydration. Microwave treatment was performed for antigen retrieval with antigen retrieval buffer. Next, all the sections were cooled at room temperature for 30 minutes. Endogenous peroxidase activity was blocked using Antibody Diluent/Block (Akoya Biosciences) for 10 minutes at room temperature. The slides were incubated for 1 hour at room temperature with primary antibodies (details of the antibodies are described in Supplementary Table S1), for 20 minutes at 37°C with secondary reagents, and for 10 minutes at room temperature with Opal working buffer. The above procedures were repeated for other antibodies, and the antibodies were removed by microwave treatment before another round of staining was performed. Nuclear staining was performed via incubation with 4',6-diamidino-2-phenylindole (DAPI; Akoya Biosciences) for 5 minutes at room temperature.

### Image analysis and spatial feature classification

The slides were visualized via the Vectra Polaris system (Akoya Biosciences), and a multispectral image of the whole slide was scanned with a 20× objective lens. Multispectral image unmixing was performed via QuPath software (version 0.5.1). Briefly, DAPI-positive cells were identified via the “cell detection” command, and each single-channel intensity threshold was selected via “object classification.” We determined positive cells with a “load classifier” (loading multiple classifiers at the same time can generate different phenotypes; for example, we can generate a CD8^+^PD-1^+^ phenotype by loading “CD8” and “PD-1”) and counting proportions by dividing the channel-positive cell counts (Supplementary Fig. S1A and S1B). All the detected cells were subsequently divided into different subgroups for further analysis, and defective TMA cores or areas with ruptured or folded tissue were reanalyzed or excluded. Spatial distance is calculated as the “distance to centroid distances 2D.” The phenotype maps were analyzed with HALO software (Indica Labs). Briefly, the spatial analysis module is a suite of four algorithms that identify the proximity and relative spatial distribution of cells and objects across whole-slide images, tissue boundaries, or serial sections. In this study, we used the infiltration analysis of the spatial analysis module to generate phenotype maps.

### Phenotype map analysis

In phenotype maps, the high CD8^+^ T-cell infiltration phenotype refers to the CD8^high^ cluster (last 25% of the CD8^+^ T-cell percentage), and the low CD8^+^ T-cell infiltration phenotype refers to the CD8^low^ cluster (least 25% of the CD8^+^ T-cell percentage).

### Clustering analysis of patients

To investigate whether spatial immune cell status is associated with the prognosis of 189 patients (Supplementary Table S2), we performed hierarchical clustering analysis. We calculated the percentage of 21 spatial immune cells among the total cells to construct the spatial feature cluster matrix for each sample. We used the get_dist function from factoextra (v1.0.7, https://github.com/kassambara/factoextra) with the maximum method to calculate the pairwise distance matrix for these patients. The hclust function from the R package stats (v4.0.2) with ward. D2 linkage was then used to perform hierarchical clustering on this matrix. The resulting dendrogram was divided into three clusters with a ratio of 20:17:152, effectively categorizing the patients into three distinct groups on the basis of their spatial immune cell statuses. The clustering results were visualized via the R package ComplexHeatmap (v2.7.9; ref. 13).

#### Prognostic analysis of clustering groups

To explore whether there are prognostic differences among the different clusters, we performed a pairwise survival difference analysis on the clustering results. Kaplan‒Meier (K‒M) curves were used to compare the prognostic differences between the groups via the survfit function of the R package (https://cran.r-project.org/web/packages/survival/index.html).

### LASSO–Cox analysis

#### Defining the training and validation cohorts

The 189 patients were randomly divided into a training set and a validation set at a 6:4 ratio by a sample function from the R package base (v4.0.2). The training set was used to determine the survival-related factors and establish the nomogram. The validation set was used to verify the nomogram. Missing values in the data for any variable were filled in with the median of that variable.

#### Construction of the prediction nomogram

The LASSO algorithm is a linear regression algorithm that can perform feature selection. A total of 21 spatial immune cells in the total immune cell population and one TNM stage feature were used to construct the prognostic model. LASSO regression was subsequently performed to further narrow down the above features to five by the cv.glmnet function of the R package glmnet (v4.1-2; ref. 14) with 10-fold cross-validation, which was determined by the minimum parameter (λ). Five features were further included in the multivariate Cox regression analysis. We found that there was no significant correlation between the variables by calculating Pearson’s correlation coefficient pairwise, which was displayed via the corrplot function from the R package corrplot (v0.90; ref. 15). A Cox proportional hazards regression analysis was then performed using the coxph function from the R package survival^4^ (v3.2-13, https://github.com/therneau/survival). The risk scores were subsequently calculated by multiplying the expression level of the spatial immune cell statuses and the multivariate Cox proportional hazards coefficient. The detailed formula was as follows:

$$\text{Risk}\;\text{Score}=\left(1.31\times {\text{CD}8}^{+}\;\text{distal}\;\text{status}\right)+\left(5.66\times {\text{CD}8}^{+}{\text{PD}‐1}^{+}\;\text{distal}\;\text{status}\right)+\left(2.15\times {\text{PD}‐1}^{+}{\text{TIM}‐3}^{+}\;\text{distal}\;\text{status}\right)+\left(-1.63\times {\text{CD}8}^{+}{\text{PD}‐1}^{+}\;\text{intratumoral}\right)+\left(0.88\times \text{TNM}\;\text{stage}\;\text{status}\right)$$

On the basis of the final results of LASSO–Cox regression, a novel nomogram including all four spatial features and one TNM stage feature was developed to predict the 1-, 3-, and 5-year OS of patients with colorectal cancer. The nomogram output was displayed via the nomogram function from the R package rms (v6.8-0, https://cran.r-project.org/web/packages/rms/index.html).

### Validation and comparison of the prediction nomogram

To measure the performance of the nomogram and compare it with the nomogram based solely on the TNM stage, both training and validation sets were used. The time-dependent ROC curve (timeROC) and the AUC were calculated via the R package timeROC (v0.4; ref. 16) to assess the predictive efficacy of the prognostic signature at 1-, 3-, and 5-year intervals. We calculated the risk scores for all patients and compared these scores between patients with different outcomes and found that the risk scores of patients with a survival outcome of 1 were significantly higher than those of patients with a survival outcome of 0. Furthermore, patients were divided into high- and low-risk groups on the basis of the median risk score. K‒M curves were used to compare the prognostic differences between the two groups via the survfit function of the R package survival (v3.2-13).

### Tumor models

The CT26 cells (RRID: CVCL_7254) were purchased from Cell Bank, Chinese Academy of Sciences, and maintained at 37°C with 5% CO_2_ in RPMI 1640 medium (HyClone) supplemented with 10% heat-inactivated FCS (Gibco), penicillin (Gibco), and streptomycin (Gibco). In this study, *Mycoplasma* detection was negative, and the cell line was proven by short tandem repeat authentication. Six to eight weeks old female BALB/c mice were used for the s.c. tumor model. Briefly, CT26 (2.5 × 10^5^) cells in 50 μL of Matrigel (Corning) were injected subcutaneously into each mouse’s right flank. Tumor volume [length × (width^2^)/2] was assessed by caliper measurements every other day, and cohorts of mice were randomized into different treatment groups according to tumor volume. Each tumor was cut in the middle equally into two parts so that each part had the same spatial features; one part was used for downstream flow cytometry analysis, and the other part was used for mIHC. All animal studies were approved by the West China Hospital Animal Ethics Committee (2020361A).

### Neutralizing antibodies

αPD-1 (RMP1-14; Cat. # BE0146, RRID: AB_10949053, Bio X Cell) and αTIM-3 (RMT3-23; Cat. # BE0115, RRID: AB_10949464, Bio X Cell) mAbs were given by i.p. injection every 4 days at 10 mg/kg to block PD-1 and TIM-3. Isotype controls (Cat. # BE0089, RRID: AB_1107769, Bio X Cell) were used at the same concentration as those in the control group.

### Flow cytometry

Single-cell suspensions were obtained by incubating minced tumor tissues with 3 mg/mL collagenase A (Roche) and 1 mg/mL DNase I (Roche) in RPMI 1640 medium at 37°C for 30 min. The resulting cell suspensions were passed through a 40-µm cell filter and treated with 1× red blood cell lysis buffer (Invitrogen). Finally, the suspension was washed with PBS and counted for flow cytometric analyses. Briefly, the cells were incubated with Fixable Viability Stain (BD) to gate viable cells. The samples were then washed with flow cytometry staining buffer (PBS containing 2% BSA), and the Fc receptor was blocked with TruStain FcX (anti-mouse CD16/32) antibody (BioLegend). The cells were incubated with cell-surface antibodies for 30 minutes at 4°C. The cells were then washed twice with flow cytometry staining buffer. Details of the antibodies used are described in Supplementary Table S3. The cells were then washed twice with permeabilization buffer and resuspended in flow cytometry staining buffer.

### Statistical analysis

We used *t* tests for measurement data and *χ*^2^ tests for count data for two variables. LASSO regression was used to filter and select variables. Multivariate Cox regression analyses were used to assess the predictive value of each risk factor. The predictive efficacy of the prognostic signatures was evaluated with the K–M curve and timeROC. Pearson correlation analysis was used to assess the correlation between two variables. The procedures involved in this study were performed with R software (v 4.0.2), and *P* < 0.05 was considered statistically significant.

### Data availability

All the data used to understand and assess the conclusions of this research are available in the main text and supplementary materials. All other data and codes are available in Code Ocean (https://codeocean.com/capsule/8443977/tree/v1).



## Results

### Patient characteristics and the mIHC panel

**Figure 1:**
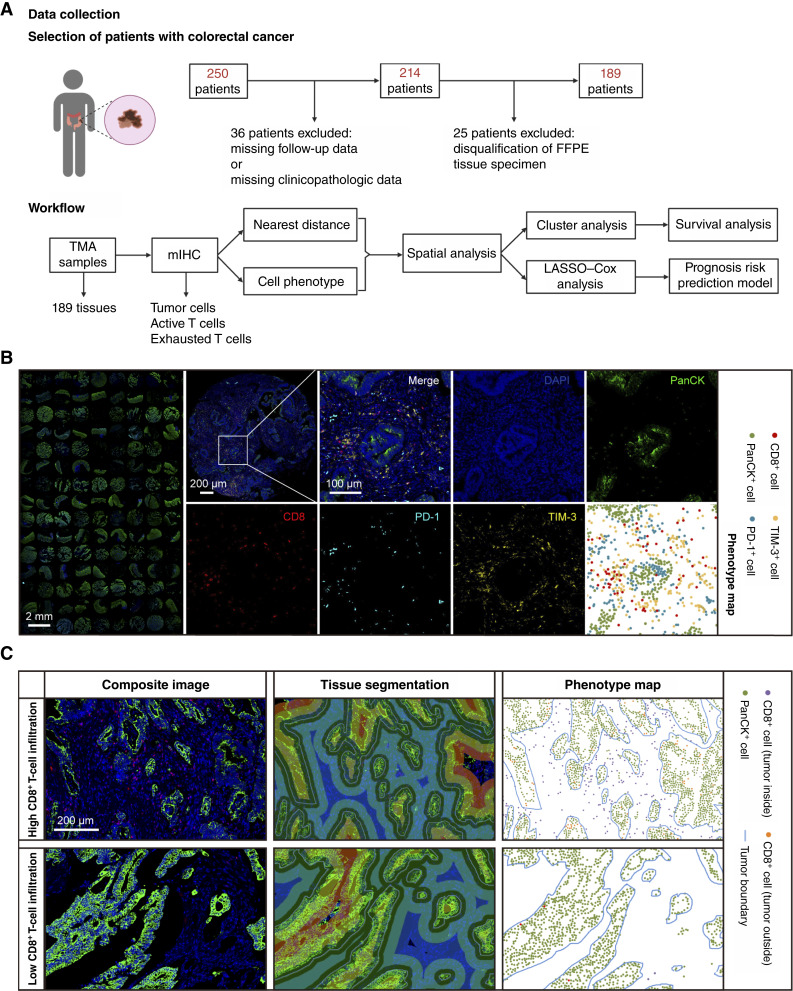
Schematic approach and different infiltration patterns of T cells among colorectal cancer tumors. **A,** Selection of patients with colorectal cancer and workflow of the present study. **B,** Representative images of multiplex immunofluorescence staining (PanCK, green; CD8, red; PD-1, cyan; and TIM-3, yellow) and phenotype map. **C,** Representative images of tissue segmentation and phenotype maps of high and low CD8^+^ T-cell infiltration. Blue lines represent tumor core area boundaries. DAPI, x; FFPE, formalin-fixed paraffin-embedded.

The cohort originally comprised 250 patients diagnosed with colorectal cancer. However, 36 patients were excluded because of incomplete follow-up data or a lack of clinicopathologic parameters. An additional 25 cases were excluded from analysis owing to disqualification of paraffin-embedded formalin-fixed tissue samples. Finally, a total of 189 patients with untreated, advanced colorectal cancer were subjected to multiplex immunofluorescence (mIHC) analysis to evaluate the TIME in human patients with colorectal cancer. Patient selection and workflow are presented in Fig. 1A. The median age of the enrolled patients was 58 years (range, 38–80), and the median overall survival (OS) time was 42 months (range, 2–85).

T-cell exhaustion is characterized by elevated expression levels of PD-1 and TIM-3 (17) on CD8^+^ T cells, and recent evidence suggests that their crosstalk regulates immunotherapy efficacy. In this study, we standardized a mIHC panel for PanCK (tumor cells), CD8 (T cells), PD-1, and TIM-3 to evaluate the infiltration of CD8^+^ cytotoxic T lymphocytes (CTL) and the status of immune checkpoints (Fig. 1B). Consistent with previous studies (18), we found a significant difference in CD8^+^ T-cell infiltration among colorectal cancer tumors (Fig. 1C; Supplementary Fig. S2). Importantly, CD8^+^ T cells also presented different spatial distribution patterns in populations with high CD8^+^ T-cell infiltration. Most of the infiltrated CD8^+^ T cells were distributed in the stroma region, and only a few infiltrated into core regions of the tumor, suggesting the necessity of deep exploration of the spatial TIME.

### Spatial heterogeneity of the immune microenvironment in human colorectal cancer

**Figure 2:**
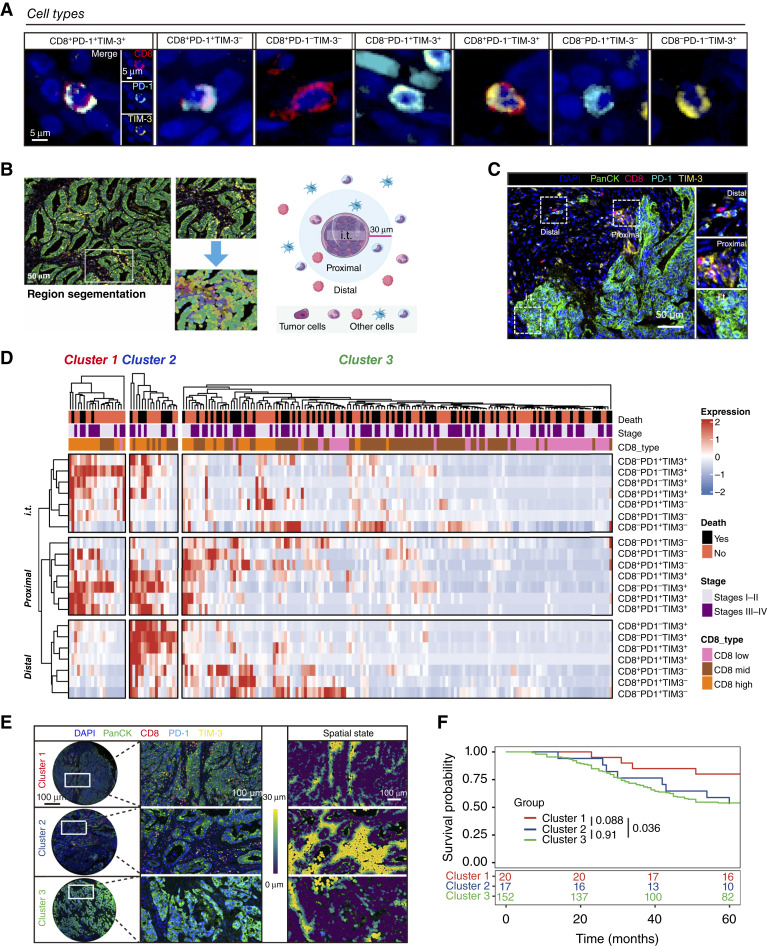
Spatial heterogeneity of the immune microenvironment correlates with patient prognosis in human colorectal cancer. **A,** Representative images of defined seven cell types (CD8^+^PD-1^+^TIM-3^+^, CD8^+^PD-1^+^TIM-3^−^, CD8^+^PD-1^−^TIM-3^−^, CD8^−^PD-1^+^TIM-3^+^, CD8^+^PD-1^−^TIM-3^+^, CD8^−^PD-1^+^TIM-3^−^, and CD8^−^PD-1^−^TIM-3^+^). **B,** Combination of the distance map and cell subtype map showed spatial infiltration patterns. Criteria of spatial division of the tumor: i.t. (within 10 μm to the tumor cell), proximal (10–30 μm to the tumor cell), and distal (more than 30 μm to the tumor cell). **C,** Representative image of microenvironment regions and different infiltration patterns of three-marker–defined CD8^+^ T cells. **D,** Hierarchical clustering of all quantified immune cells and their spatial phenotypes. Heatmap represents *z*-score–normalized data; red color represents expression above the mean, blue color represents expression below the mean, and white color represents the mean. **E,** Representative image and spatial state of three subtypes: cluster 1, high-infiltrated; cluster 2, medium-infiltrated; and cluster 3, low-infiltrated, characterized by infiltration of CTLs; blue color represents cells at a distance of 0 μm from the tumor cells, and yellow color represents cells at a distance of 30 μm from the tumor cells. **F,** K–M curve (log-rank test) survival plots depict the OS in the unsupervised clusters. DAPI,x; mid, medium.

The numbers of cells were calculated via cell segmentation, and a total of seven types of cells were defined by diverse expression patterns of three markers: CD8^+^PD-1^+^TIM-3^+^, CD8^+^PD-1^+^TIM-3^−^, CD8^+^PD-1^−^TIM-3^−^, CD8^−^PD-1^+^TIM-3^+^, CD8^+^PD-1^−^TIM-3^+^, CD8^−^PD-1^+^TIM-3^−^, and CD8^−^PD-1^−^TIM-3^+^ (Fig. 2A). The spatial patterns of immune infiltration were defined as intratumoral, proximal, and distal regions. We defined cells that were distributed outside the 30 μm radius from the tumor cell as those belonging to the distal region. The proximal region was the region in which cells were distributed within a 30 μm radius from the nuclear center of any given tumor cell, and the i.t. region was defined as the region in which cells infiltrated the tumor epithelium (Fig. 2B). Then, we combined the spatial phenotype and cell segmentation phenotype and formed the final infiltration phenotype. The representative distributions of different CD8^+^ T-cell phenotypes in the distal, proximal, and i.t. regions are shown in Fig. 2C.

To explore the heterogeneity in the spatial distribution of different subsets, we performed hierarchical clustering for all patients with all studied cell types (Fig. 2D). Patients with colorectal cancer were clustered into three subtypes on the basis of previous studies: high-infiltrated (cluster 1 with 20 patients), medium-infiltrated (cluster 2 with 17 patients), and low-infiltrated (cluster 3 with 152 patients) subtypes characterized by infiltration of CTLs (Fig. 2E).

Briefly, there was no difference in the clinicopathologic characteristics among the three clusters (Supplementary Table S2). The total cell infiltration data are shown in Supplementary Fig. S3. The trends of the presence of each cell type were similar in the stromal, proximity, and i.t. regions. We subsequently assessed the associations between the presence of T cells and spatial clusters (Supplementary Fig. S3A–S3C). Cluster 3 had the lowest immune cell infiltration, and the cell count of infiltrated cells was significantly lower in every region and every cell type. T cells presented different trends in the distal region compared with the proximal and i.t. regions. For example, in the proximal and i.t. regions, the number of CD8^+^ T cells in cluster 1 was greater than that in cluster 2, yet cluster 1 had fewer CD8^+^ T cells in the distal region, indicating that cluster 1, as the high-infiltrated cluster, had more T cells infiltrating into the core area of the tumor (Fig. 2E).

Notably, K–M curves revealed that survival was significantly longer among patients in cluster 1 than among those in clusters 2 and 3, and these results are consistent with previous studies (19) and demonstrate the benefits of highly infiltrated T cells in tumors, especially in the i.t. region. However, there was no survival difference between clusters 2 and 3, indicating a limitation of prognostic prediction methods based on traditional T-cell infiltration analysis and simple spatial clustering (Fig. 2F).

### Spatial contexture of immune checkpoints predicts OS independent of standard TNM stages

**Figure 3:**
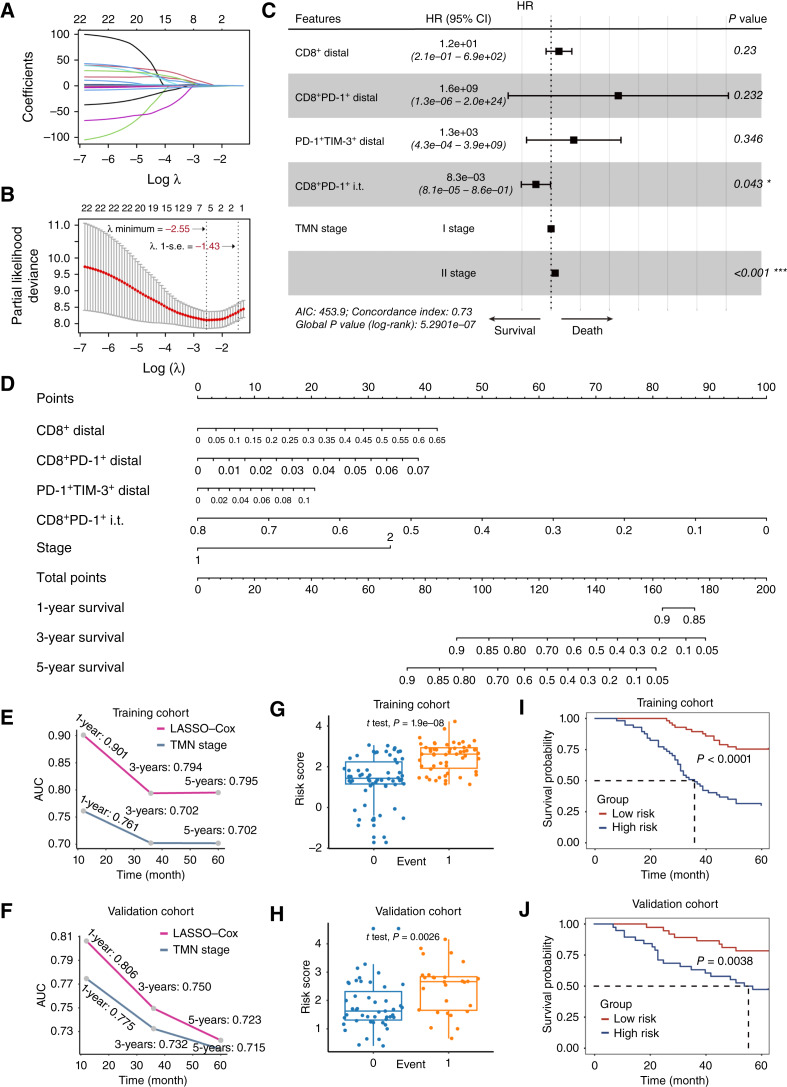
Construction of LASSO–Cox regression model based on immune spatial features. **A,** LASSO model coefficient profiles of significant features with the top included times in the training cohort. **B,** Parameter selection in the LASSO model, by 10-fold cross-validation via minimum criteria *λ* = 0.0779, was chosen in the training cohort. **C,** Multivariate Cox regression analysis for the prognosis-related genes. **D,** The nomogram predicts the OS of patients with colorectal cancer by incorporating multiple immunologic and clinical factors. The variables included are CD8^+^ T cells at distal sites, CD8^+^PD-1^+^ T cells at distal sites, PD-1^+^TIM-3^+^ T cells at distal sites, CD8^+^PD-1^+^ T cells at i.t. sites, and clinical stage. Each variable is assigned a score, which is then summed to provide a total score. This total score is used to estimate the probabilities of 1-, 3-, and 5-year survival. **E** and **F,** Comparison of the AUC values between the LASSO–Cox model and TNM stage model at 1, 3, and 5 years in the training cohort (*n* = 114) and the validation cohort (*n* = 75). **G** and **H,** Comparison of the risk score between deceased patients (event = 1) and living patients (event = 0) in the training cohort (*n* = 114) and the validation cohort (*n* = 75). Data were determined by a two-tailed unpaired *t* test. **I** and **J,** K–M curve survival analysis stratification based on a median risk score cutoff in the training and validation cohorts. CI, confidence interval; min, minimum; y, year; ys, years.

To examine whether spatial distance might have a promising value for OS, we randomly divided all patients into a training cohort set (*n* = 114) and a validation cohort set (*n* = 75) at a 6:4 ratio. We found that there were no significant differences in clinicopathologic characteristics between these two cohorts (Supplementary Table S4). Next, a total of 21 spatial immune cell statuses were collected for further analysis (Supplementary Table S5), which revealed no differences between the two cohorts, indicating random and reasonable grouping (Supplementary Fig. S4A–S4C). The LASSO–Cox model was used to establish the prognostic scoring system in the training cohort. First, we used LASSO regression to screen parameters, and then the variation characteristics of the coefficient of these variables were shown via LASSO regression (Fig. 3A). The 10-fold cross-validation method was applied to the iterative analysis. Our model achieved its best performance with a minimum number of variables when *λ* was chosen as 0.0779 (Log*λ* = −2.55; Fig. 3B). Finally, through the calculation and deformation of the LASSO model, we obtained 4 features (CD8^+^ distal, CD8^+^PD-1^+^ distal, PD-1^+^TIM-3^+^ distal, and CD8^+^PD-1^+^ i.t.) from 21 types of immune cell density and tumor TNM stages. In addition, no significant correlation was found among those factors (Supplementary Fig. S5). The Cox regression model was then performed depending on the features mentioned above. A forest plot revealed that these factors were associated with the prognosis of patients with colorectal cancer (Fig. 3C).

The forest plot suggested that CD8^+^ distal, CD8^+^PD-1^+^ distal, PD-1^+^TIM-3^+^ distal, and advanced primary tumor stages were negative factors for prognosis, whereas CD8^+^PD-1^+^ i.t. factors were favorable prognostic factors. A formula was also constructed to obtain the score for each patient on the basis of the expression level of the above-selected features, in which the risk score = (1.31 × CD8^+^ distal status) + (5.66 × CD8^+^PD-1^+^ distal status) + (2.15 × PD-1^+^TIM-3^+^ distal status) + (−1.63 ×CD8^+^PD-1^+^ i.t.*) + (0.88 × TNM stage status*). In this formula, the TNM stage is divided into I–II (status 1) and III–IV groups (status 2).

To provide a simple vision of our risk score model, we constructed a nomogram as a quantitative tool to predict the 1-, 3-, and 5-year mortality rates of patients with colorectal cancer (Fig. 3D). The nomogram integrates factors such as immune infiltration and TNM stages, allowing clinicians and pathologists to easily compute the risk score for each patient. A comparison of the AUC values between our model and TNM stages only supported the better clinical prediction of our method. Furthermore, the AUC values decreased as the survival time increased, regardless of our method or the TNM stage, whereas the values obtained via our method remained at approximately 0.8, indicating good prediction ability (Fig. 3E and F). To test the accuracy of our risk score system, a comparison was performed between deceased patients (event = 1) and living patients (event = 0), and deceased patients presented markedly higher risk scores, which substantiates the reliability of our system (Fig. 3G and H).

Finally, patients were categorized into high-risk or low-risk groups on the basis of a median risk score cutoff. (OS analysis revealed that patients with lower risk scores had a better prognosis in both the training and validation cohorts, confirming the validity of the LASSO–Cox regression risk score model (Fig. 3I and J).

Above all, we verified the potential impact of spatial immune cell distribution on OS in patients, identified key immune features that influence prognosis, and developed a scoring system for patients with colorectal cancer via the LASSO–Cox model, which showed better precision than the previous method of TNM staging.

### TIM-3 and PD-1 dual blockade improves antitumor effects through the inhibition of T-cell exhaustion

**Figure 4:**
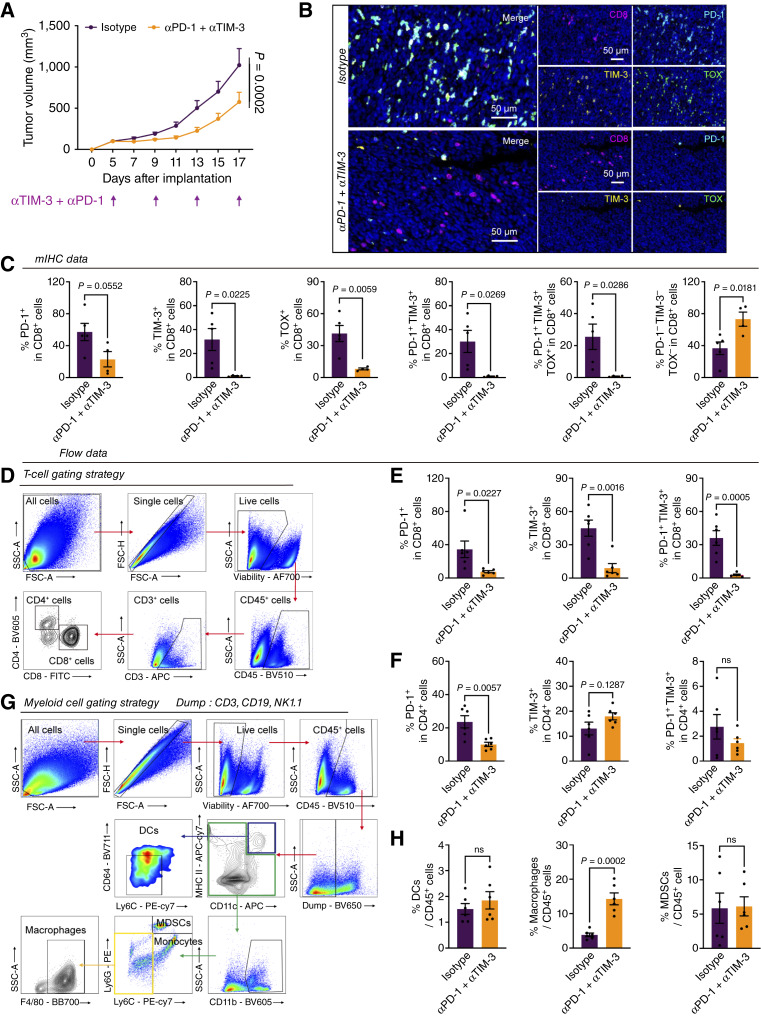
TIM-3 and PD-1 dual blockade reverses T-cell exhaustion and enhances the antitumor immune response. **A,** Tumor volume. A measure of 2.5 × 10^5^ CT26 cells were injected subcutaneously into BALB/C mice, followed by treatment with isotype or αPD-1 (RMP1-14) and αTIM-3 (RMT3-23) antibodies (10 mg/kg) every 4 days (isotype: *n* = 6 mice per arm; αPD-1 + αTIM-3: *n* = 7 mice per arm). Data are presented as the mean ± SEM and determined by two-way ANOVA. **B,** Representative images of multiplex immunofluorescence staining (CD8, red; Ki67, white; PD-1, cyan; TIM-3, yellow; and TOX, green) in isotype or αPD-1 + αTIM-3 treatment tumor. **C,** mIHC quantification of PD-1^+^CD8^+^ T cells, TIM-3^+^CD8^+^ T cells, TOX^+^CD8^+^ T cells, PD-1^+^TIM-3^+^CD8^+^ T cells, PD-1^+^TIM-3^+^TOX^+^CD8^+^ T cells, and PD-1^−^TIM-3^−^TOX^−^CD8^+^ T cells as a percentage of CD8^+^ cells. Data are presented as the mean ± SEM and determined by a two-tailed unpaired *t* test. **D,** T-cell panel gating strategy. **E,** FACS quantification of PD-1^+^CD8^+^ T cells, TIM-3^+^CD8^+^ T cells, and PD-1^+^TIM-3^+^CD8^+^ T cells as a percentage of CD8^+^ T cells. Data are presented as the mean ± SEM and determined by a two-tailed unpaired *t* test. **F,** FACS quantification of PD-1^+^CD4^+^ T cells, TIM-3^+^CD4^+^ T cells, and PD-1^+^TIM-3^+^CD4^+^ T cells as a percentage of CD4^+^ cells. Data are presented as the mean ± SEM and determined by a two-tailed unpaired *t* test. **G,** Myeloid cell panel gating strategy. **H,** FACS quantification of dendritic cells, macrophages, and myeloid-derived suppressor cells as a percentage of CD45^+^ cells in the αPD-1 + αTIM-3 experiment. Data are presented as the mean ± SEM and determined by a two-tailed unpaired *t* test. DC, dendritic cell; MDSC, myeloid-derived suppressor cell.

On the basis of our previous analysis, PD-1 and TIM-3 have shown potential in prognosis prediction; thus, their further influence on clinical treatment is worth exploring. Consequently, a combination blockade of PD-1 and TIM-3 was performed. The application of αPD-1 and αTIM-3 antibodies in CT26 mouse models of human colorectal cancer notably suppressed tumor growth. (Fig. 4A). In line with our expectations, we found that both PD-1 and TIM-3 expression was significantly decreased in the combination treatment group (Supplementary Fig. S6A and S6B). To better explore the changes in the TIME after αPD-1 and αTIM-3 antibody treatment, a mIHC panel (CD8, Ki67, PD-1, TIM-3, and TOX) was designed to analyze the state of CD8^+^ T cells in tumors from both the control and treatment groups (Fig. 4B). Treatment with the combination of αPD-1 and αTIM-3 decreased the proportion of exhausted CD8^+^ T cells (PD-1^+^CD8^+^, TIM-3^+^CD8^+^, and TOX^+^CD8^+^). Notably, the proportions of terminally exhausted CD8^+^ T cells (PD-1^+^TIM-3^+^CD8^+^ T cells and PD-1^+^TIM-3^+^TOX^+^CD8^+^ T cells) were also significantly lower in the combination treatment group than in the control group. In contrast, we observed a significant increase in functional CD8^+^ T cells (PD-1^−^TIM-3^−^TOX^−^CD8^+^) in tumors from combination-treated mice (Fig. 4C). In addition, a cytotoxic mIHC panel (CD8 and granzyme B) was used to assess the state of CD8^+^ T cells, and we found that more cytotoxic CD8^+^ T cells (granzyme B^+^CD8^+^) were identified in the combination treatment group (Supplementary Fig. S6C and S6D). The results of flow cytometry revealed a similar trend of reversing T-cell exhaustion not only in CD8^+^ T cells but also in CD4^+^ T cells (Fig. 4D–F). Moreover, the combination treatment also increased the infiltration of macrophages, whereas no changes in myeloid-derived suppressor cells were observed, indicating the possible antitumor effect of macrophages (Fig. 4G and H).

Taken together, these data demonstrated that dual targeting of PD-1 and TIM-3 inhibited tumor progression and reduced T-cell exhaustion.

## Discussion

Despite the use of multimodal treatments, including neoadjuvant therapy, surgery, and postoperative adjuvant therapy, the overall prognosis of patients with colorectal cancer is still unsatisfactory (1, 20). In the present study, we demonstrated that the spatial context of PD-1 and TIM-3 successfully predicted the OS of patients with colorectal cancer independent of TNM stage. Dual targeting of PD-1 and TIM-3 in mouse tumor models inhibited tumor progression and reduced T-cell exhaustion. Our findings could pave the way for the development of novel therapeutic strategies aimed at modulating the spatial distribution of immune checkpoints to enhance antitumor immune responses.

Recently, classifications based on the TME have emerged; for example, three distinct lymphocyte infiltration modules have been identified as follows (21–23): (i) the inflamed/infiltrated phenotype, in which CD8^+^ T cells infiltrate the tumor epithelium; (ii) the excluded phenotype, in which infiltrating CD8^+^ T cells accumulate in the tumor stroma rather than the tumor epithelium; and (iii) the immune desert/ignored phenotype, in which CD8^+^ T cells are either absent or present in very low numbers. Consistently, when the patients with colorectal cancer in this work were clustered into three subtypes, high-infiltrated (cluster 1), medium-infiltrated (cluster 2), and low-infiltrated (cluster 3), which were characterized by infiltration of CTLs according to previous studies, cluster 1 had a significantly better prognosis than clusters 2 and 3. The Immunoscore (Veracyte) assay is primarily based on the density and location of cytotoxic T cells within the TME and surrounding tissues (24). The advantages of the Immunoscore assay include its simplicity and proven effectiveness in various studies. However, its main drawback is that, although it considers the spatial distribution of cytotoxic T cells, neglecting the immune status of these cells could lead to a loss of prognostic information.

The spatial immune microenvironment refers to the distribution and interactions of immune cells within the TME, including tumor-infiltrating lymphocytes, tumor-associated macrophages (TAM), and other immune and nonimmune cells (22, 25–28). Understanding the spatial immune microenvironment is important in the context of cancer, as it plays a crucial role in shaping the immune response to cancer and impacting the efficacy of immunotherapies (29, 30). To the best of our knowledge, little is known about specific changes in the diversity and functional status of tumor-infiltrating lymphocytes within the spatial setting of the TME in relation to the survival and therapeutic response of patients with colorectal cancer. In this study, we established a risk score model based on the LASSO–Cox regression method, which included immune cell status, immune cell spatial distribution, and clinicopathologic stage. These results indicate that i.t. PD-1^+^TIM-3^+^ status, i.t. CD8^+^PD-1^+^ status, proximal PD-1^+^TIM-3^+^ status, proximal PD-1^+^ status, and distal CD8^+^TIM-3^+^ status are important spatial factors related to patient survival. Furthermore, compared with a single TNM stage, our model composed of multiple immune features showed better accuracy of prognosis in patients with colorectal cancer.

LASSO–Cox regression is a commonly used statistical method that effectively performs feature selection on high-dimensional data, identifying the variables with the most significant impact on prognosis. By using LASSO–Cox regression, we identified a model tailored to our experimental technique that can predict patient prognosis effectively. Our model offers a novel approach to prognostic analysis on the basis of spatial information. Building on the foundation of the Immunoscore assay, our model focuses on the spatial distribution of immune cells with distinct immune statuses within different regions of the tumor. This allows our model to reflect more comprehensively the immunologic characteristics of the TME, thereby offering a more precise prognostic analysis.

Exhausted T cells have been shown with elevated expression levels of inhibitory receptors such as PD-1, Lag3 and TIM-3. Increasing evidence suggests that their crosstalk regulates T-cell exhaustion and immunotherapy efficacy. TIM-3 is a transmembrane protein that is expressed on the surface of various immune cells, including T cells and NK cells (31, 32). It was initially identified as being selectively expressed on Th1 and Tc1 cytotoxic T cells, which are involved in cell-mediated immunity and play crucial roles in fighting cancer and infections (31, 33). In recent years, TIM-3 has emerged as a promising target for cancer immunotherapy, and anti-TIM-3 therapies are being explored as a means of reversing T-cell exhaustion and restoring antitumor immunity (32, 34, 35). Studies have shown that the expression of TIM-3 on CD8^+^ T cells is associated with the disease stage in human colorectal cancer and that TIM-3 blockade improves antitumor responses (36–38). Consistent with these findings, we demonstrated that inhibition of TIM-3 by mAb therapy slows tumor progression in a CT26 mouse model of human colorectal cancer and that this antitumor effect is achieved by reversing T-cell exhaustion. Furthermore, our findings have shown that dual targeting of the TIM-3 and PD-1 pathways reduces T-cell exhaustion and restores better antitumor responses. Taken together, these studies provide promising evidence that targeting the TIM-3 and PD-1 pathways may represent a promising strategy for cancer immunotherapy.

Emerging evidence shows that an increase in T-cell and myeloid cell niches within posttreatment tumors indicates a better response and improved survival. A recent study revealed that the presence of M1 TAMs is associated with strong CD8^+^ T-cell tumor infiltration and better survival outcomes (39). Another study suggested that high numbers of TAMs and tumor-associated dendritic cells are related to better survival in patients with colorectal cancer (40, 41). Consistent with those findings, we also observed niches of macrophages and dendritic cells that might crosstalk with T cells in tumors from patients with better survival. Interestingly, there was a significant increase in macrophages and a slight increase in the percentage of M2 macrophages (CD206^+^MHCII^+^ macrophages) in tumors from the combination-treated mice, suggesting that these macrophages are more M1-like (Supplementary Fig. S6E). More detailed mechanisms need to be further investigated.

In summary, our data demonstrate the importance of the spatial status of infiltrated immune cells in human colorectal cancer, with an emphasis on spatial profiling of the exhausted state of immune cells in the TME. In addition, we illuminate the key roles of TIM-3 and PD-1 in cancer immunotherapy. These results highlight the importance of considering the spatial distribution of immune cells and the underlying mechanisms of immune exhaustion in the design of cancer immunotherapies.

## Supplementary Material

Figure S1 Workflow of mIHC analysis

Figure S2 Patients were divided into three groups based on the percentages of CD8+ T cells

Figure S3 Comparative analysis of CD8+ cell subtypes distribution across spatial clusters in unsupervised groupings

Figure S4 Analysis of immune cell statuses across different tumor locations in training and validation cohorts.

Figure S5 Pearson correlation of screened factors

Figure S6 Immune cell status changes after αPD-1 + αTIM-3 treatment

Table S1 Abs for mIHC staining

Table S2 Clinical characteristics of patients from TMA study

Table S3 Abs for FACS analysis

Table S4 Clinical characteristics of training and validation cohorts

Table S5 Spatial phenotypes of CD8+ T cells
