# Supplementary material for: Spatial Context of Immune Checkpoints as Predictors of Overall Survival in Patients with Resectable Colorectal Cancer Independent of Standard Tumor–Node–Metastasis Stages
Source: Cancer Res Commun. 2024 Nov 26;4(11):3025–35. doi: 10.1158/2767-9764.CRC-24-0270 (PMC11589669; doi:10.1158/2767-9764.CRC-24-0270)
Supplement: Figure S1 — Workflow of mIHC analysis [file crc-24-0270_figure_s1_suppsf1.pdf]

Figure S1

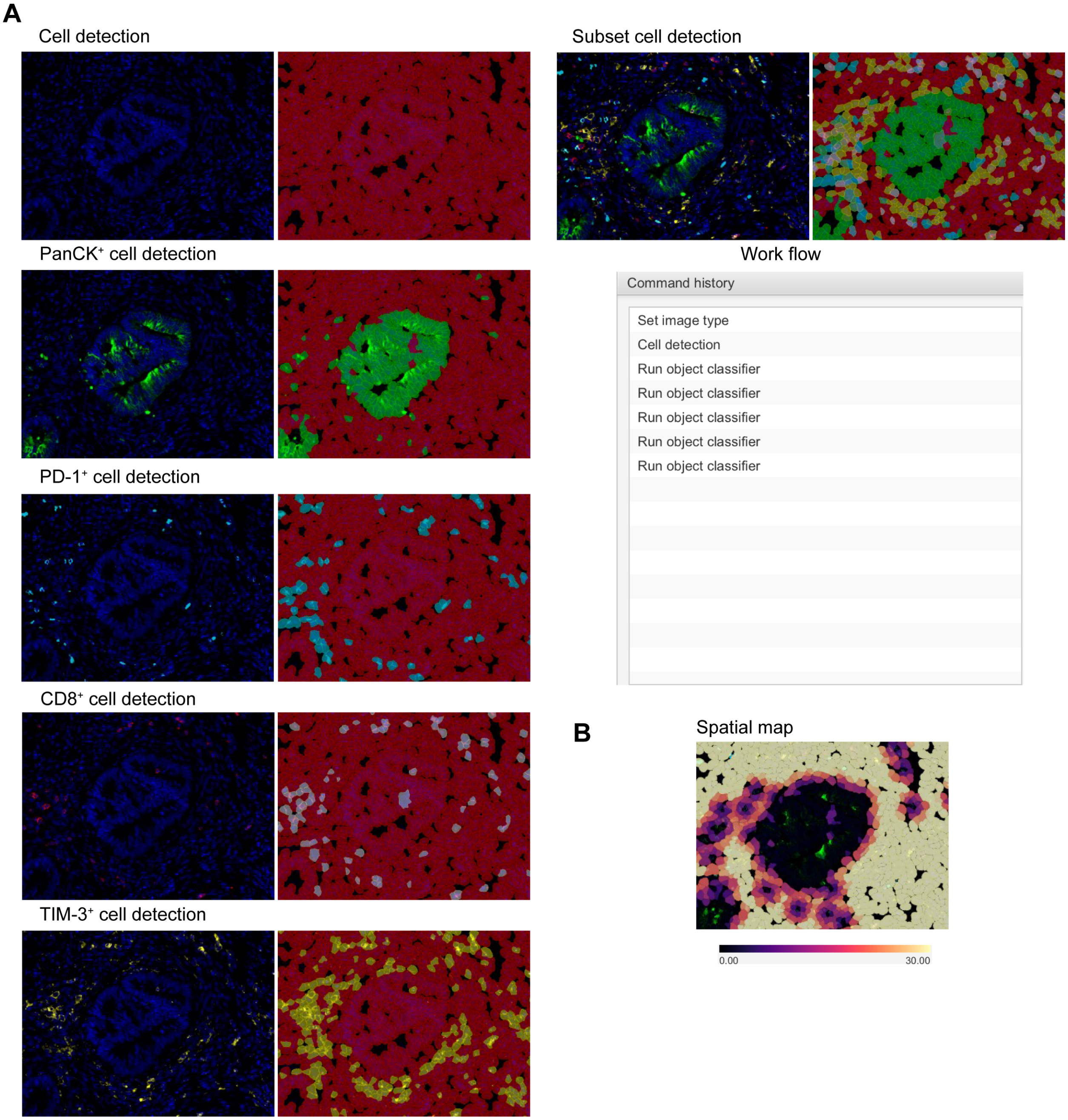

Figure S1

Workflow of mIHC analysis. A, Positive cells were determined using ‘Load classifier’ module of Qupath. **B,** Representative image of spatial analysis results.
