# Supplementary material for: Spatial Context of Immune Checkpoints as Predictors of Overall Survival in Patients with Resectable Colorectal Cancer Independent of Standard Tumor–Node–Metastasis Stages
Source: Cancer Res Commun. 2024 Nov 26;4(11):3025–35. doi: 10.1158/2767-9764.CRC-24-0270 (PMC11589669; doi:10.1158/2767-9764.CRC-24-0270)
Supplement: Figure S2 — Patients were divided into three groups based on the percentages of CD8+ T cells [file crc-24-0270_figure_s2_suppsf2.pdf]

Figure S2

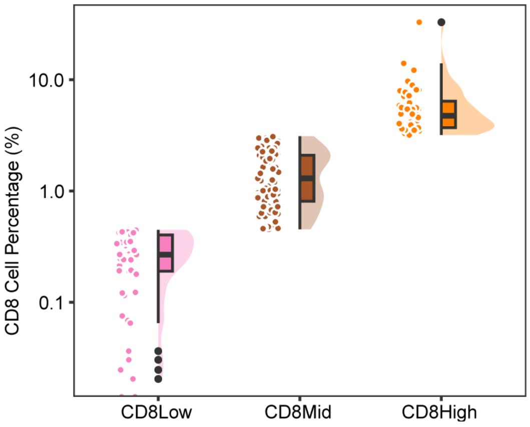

Figure S2

Patients were divided into three groups based on the percentages of CD8^{+} T cells. Patients' clusters: CD8^{low}, least 25% of CD8^{+} T cells percentage; CD8^{mid}, 25%-75% of CD8^{+} T cells percentage; CD8^{high}, last 25% of CD8^{+} T cells percentage.
