# Supplementary material for: Spatial Context of Immune Checkpoints as Predictors of Overall Survival in Patients with Resectable Colorectal Cancer Independent of Standard Tumor–Node–Metastasis Stages
Source: Cancer Res Commun. 2024 Nov 26;4(11):3025–35. doi: 10.1158/2767-9764.CRC-24-0270 (PMC11589669; doi:10.1158/2767-9764.CRC-24-0270)
Supplement: Figure S3 — Comparative analysis of CD8+ cell subtypes distribution across spatial clusters in unsupervised groupings [file crc-24-0270_figure_s3_suppsf3.pdf]

Figure S3

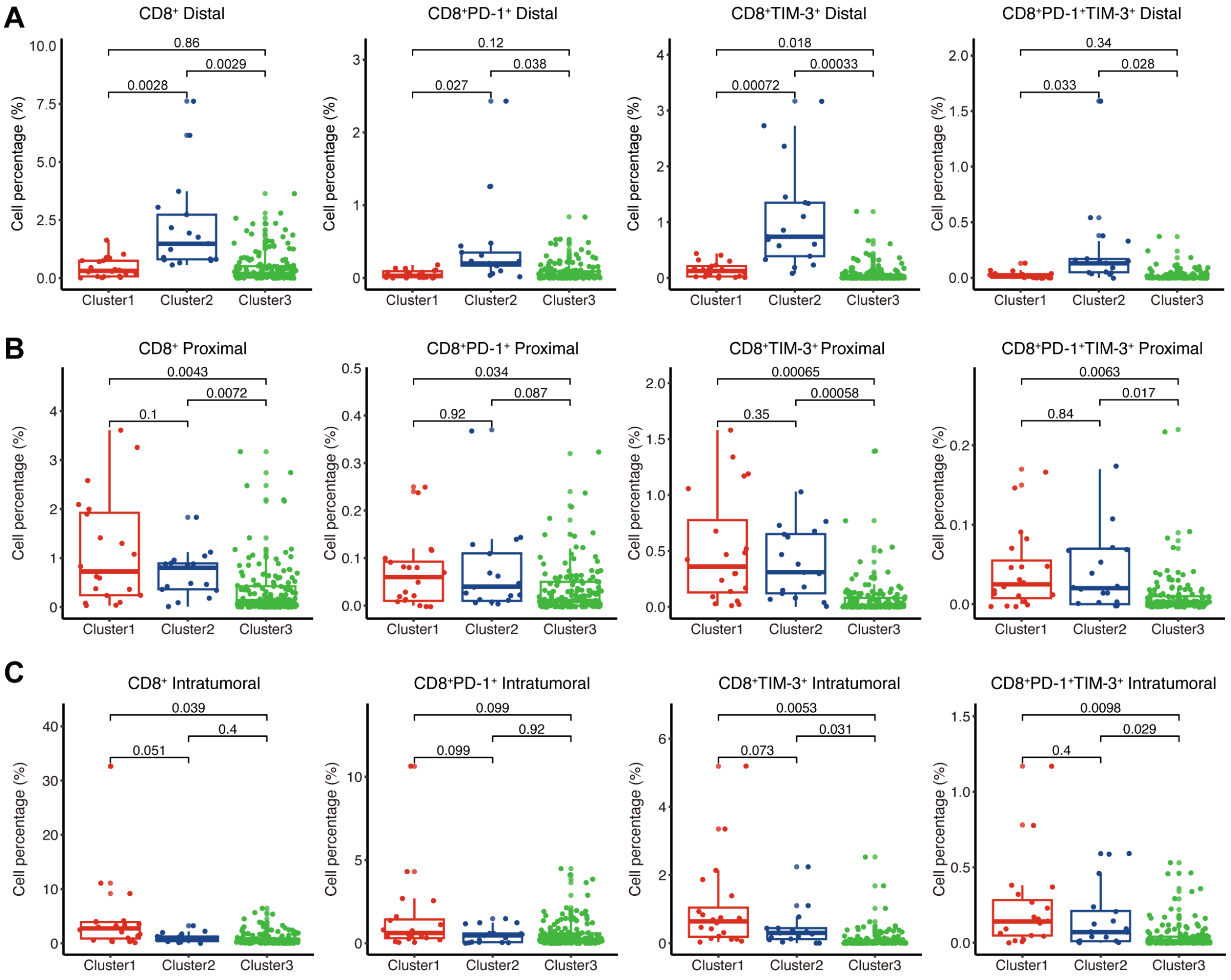

Figure S3

Comparative analysis of CD8$^+$ cell subtypes distribution across spatial clusters in unsupervised groupings. A, Boxplots depicting the proportions of distal CD8$^+$ cells subtype in the groups with unsupervised clusters. Data are determined by wilcoxon test. **B,** Boxplots depicting the proportions of proximal CD8$^+$ cells subtype in the groups with unsupervised clusters. Data are determined by wilcoxon test. **C,** Boxplots depicting the proportions of intratumoral CD8$^+$ cells subtype in the groups with unsupervised clusters. Data are determined by wilcoxon test.
