# Supplementary material for: Spatial Context of Immune Checkpoints as Predictors of Overall Survival in Patients with Resectable Colorectal Cancer Independent of Standard Tumor–Node–Metastasis Stages
Source: Cancer Res Commun. 2024 Nov 26;4(11):3025–35. doi: 10.1158/2767-9764.CRC-24-0270 (PMC11589669; doi:10.1158/2767-9764.CRC-24-0270)
Supplement: Figure S4 — Analysis of immune cell statuses across different tumor locations in training and validation cohorts. [file crc-24-0270_figure_s4_suppsf4.pdf]

Figure S4

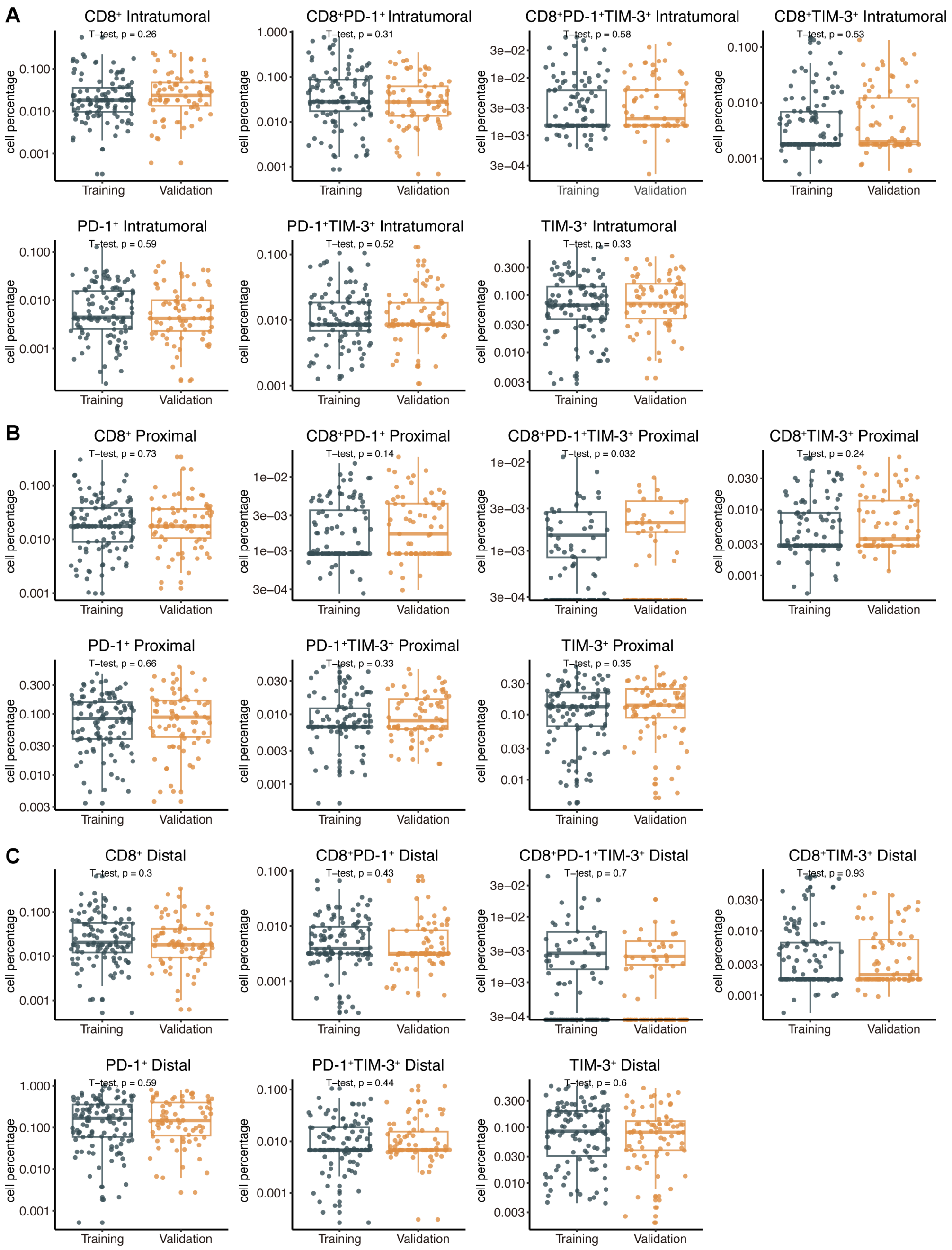

Figure S4

analysis of immune cell statuses across different tumor locations in training and validation cohorts. **A,** Comparison of 7 intratumoral immune cell statuses between training cohort and validation cohort. Data are presented as mean ± s.e.m, and determined by two-tailed unpaired t-test. **B,** Comparison of 7 proximal immune cell statuses between training cohort and validation cohort. Data are presented as mean ± s.e.m, and determined by two-tailed unpaired t-test. **C,** Comparison of 7 distal immune cell statuses between training cohort and validation cohort. Data are presented as mean ± s.e.m, and determined by two-tailed unpaired t-test.
