# Supplementary material for: Spatial Context of Immune Checkpoints as Predictors of Overall Survival in Patients with Resectable Colorectal Cancer Independent of Standard Tumor–Node–Metastasis Stages
Source: Cancer Res Commun. 2024 Nov 26;4(11):3025–35. doi: 10.1158/2767-9764.CRC-24-0270 (PMC11589669; doi:10.1158/2767-9764.CRC-24-0270)
Supplement: Figure S5 — Pearson correlation of screened factors [file crc-24-0270_figure_s5_suppsf5.pdf]

Figure S5

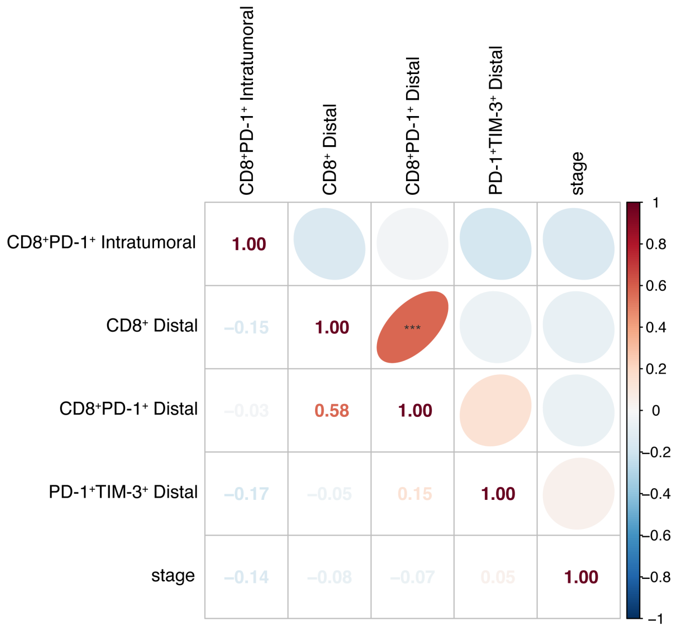

Figure S5

Pearson correlation of screened factors. The heatmap displayed a Pearson correlation coefficient matrix for five variables selected via lasso regression. Red denoted a positive correlation and blue indicated a negative correlation.
