# Supplementary material for: Spatial Context of Immune Checkpoints as Predictors of Overall Survival in Patients with Resectable Colorectal Cancer Independent of Standard Tumor–Node–Metastasis Stages
Source: Cancer Res Commun. 2024 Nov 26;4(11):3025–35. doi: 10.1158/2767-9764.CRC-24-0270 (PMC11589669; doi:10.1158/2767-9764.CRC-24-0270)
Supplement: Figure S6 — Immune cell status changes after αPD-1 + αTIM-3 treatment [file crc-24-0270_figure_s6_suppsf6.pdf]

Figure S6

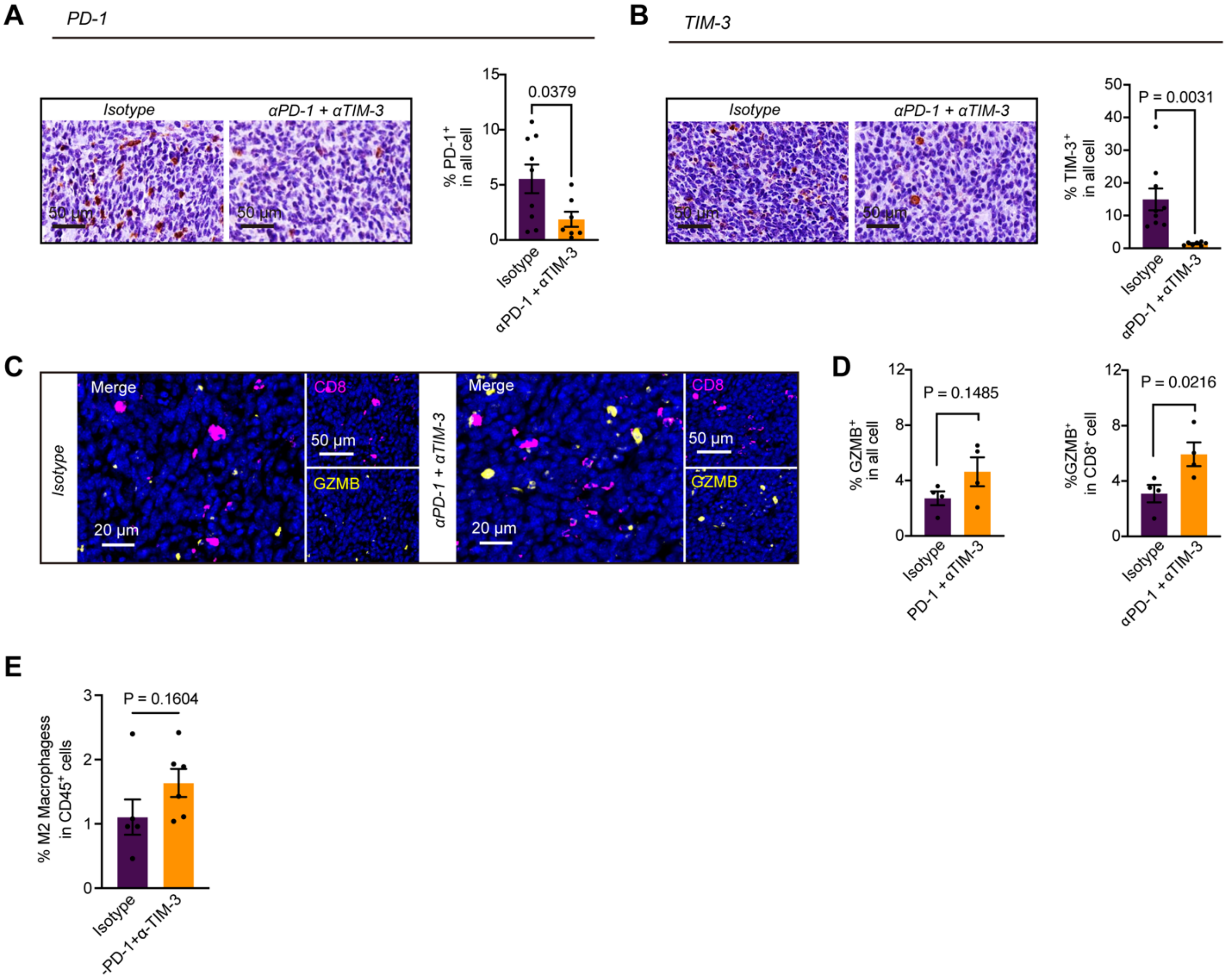

Figure S6

Immune cell status changes after αPD-1 + αTIM-3 treatment. A, Representative immunohistochemistry and quantification for PD-1 protein expression levels in CT26 tumors treated with isotype or αPD-1 + αTIM-3. Data are presented as mean ± s.e.m, and determined by two-tailed unpaired t-test. **B,** Representative immunohistochemistry and quantification for TIM-3 protein expression levels in CT26 tumors treated with isotype or αPD-1 + αTIM-3. Data are presented as mean ± s.e.m, and determined by two-tailed unpaired t-test. **C,** Representative image of multiplex immunofluorescence staining (CD8, red; GZMB, yellow) in isotype or αPD-1 + αTIM-3 treatment tumor. **D,** mIHC quantification of $GZMB^+$ cells, $GZMB^+$ T cells as a percentage of all cells or $CD8^+$ cells. Data are presented as mean ± s.e.m, and determined by two-tailed unpaired t-test. **E,** M2 macrophages are shown as a percentage of $CD45^+$ cells. The data are presented as the mean ± s.e.m, and were analyzed by two- tailed unpaired t-tests.
